# A Feedback Loop Formed by ATG7/Autophagy, FOXO3a/miR-145 and PD-L1 Regulates Stem-Like Properties and Invasion in Human Bladder Cancer

**DOI:** 10.3390/cancers11030349

**Published:** 2019-03-12

**Authors:** Junlan Zhu, Yang Li, Yisi Luo, Jiheng Xu, Huating Liufu, Zhongxian Tian, Chao Huang, Jingxia Li, Chuanshu Huang

**Affiliations:** Nelson Institute of Environmental Medicine, New York University School of Medicine, New York, NY 10010, USA; junlanzhu@163.com (J.Z.); li_yangvip@126.com (Y.L.); 15726816641@163.com (Y.L.); Jiheng.xu@nyulangone.org (J.X.); liufu624@126.com (H.L.); tianzhongxian1990@163.com (Z.T.); huangchao1009@163.com (C.H.); jingxiali@hotmail.com (J.L.)

**Keywords:** ATG7, PD-L1, stem-like property, invasion, human bladder cancer

## Abstract

Programmed cell death protein 1 (PD-1) and its ligand PD-L1 blockade have been identified to target immune checkpoints to treat human cancers with durable clinical benefit. Several studies reveal that the response to PD-1-PD-L1 blockade might correlate with PD-L1 expression levels in tumor cells. However, the mechanistic pathways that regulate PD-L1 protein expression are not understood. Here, we reported that PD-L1 protein is regulated by ATG7-autophagy with an ATG7-initiated positive feedback loop in bladder cancer (BC). Mechanistic studies revealed that ATG7 overexpression elevates PD-L1 protein level mainly through promoting autophagy-mediated degradation of FOXO3a, thereby inhibiting its initiated miR-145 transcription. The lower expression of miR-145 increases *pd-l1* mRNA stability due to the reduction of its direct binding to 3′-UTR of *pd-l1* mRNA, in turn leading to increasing in *pd-l1* mRNA stability and expression, and finally enhancing stem-like property and invasion of BC cells. Notably, overexpression of PD-L1 in ATG7 knockdown cells can reverse the defect of autophagy activation, FOXO3A degradation, and miR-145 transcription attenuation. Collectively, our results revealed a positive feedback loop to promoting PD-L1 expression in human BC cells. Our study uncovers a novel molecular mechanism for regulating *pd-l1* mRNA stability and expression via ATG7/autophagy/FOXO3A/miR-145 axis and reveals the potential for using combination treatment with autophagy inhibitors and PD-1/PD-L1 immune checkpoint blockade to enhance therapeutic efficacy for human BCs.

## 1. Introduction

Bladder cancer (BC) is the fourth most common cancer in men and the fifth most common malignancy in the US [1]. BC is categorized as either non-muscle invasive or muscle invasive, and this differentiation is key to treatment planning and prognosis [2]. Survival of patients with muscle-invasive bladder cancer (MIBC) is poor, with only 35% survival at five years in lymph node-positive tumors [3]. There have been great improvements in BC diagnosis and treatment nowadays, due to the evolution of imaging, the introduction of new diagnostic modalities, the urinary tumor markers and the improvement of the surgical skills and techniques, as well as the upgraded role of chemo- and immunotherapy. However, BC remains a major clinical concern because of its great recurrence rate and, in many cases, progression propensity [4]. Cancer stem cells (CSCs) is a subpopulation of tumor cells that are enriched for tumorigenic property and can regenerate the heterogeneity of the original tumor in immunocompromised mice [5,6]. Growing evidence suggests that basal muscle-invasive BC comprises a small population of CSCs, which is thought to be associated with BC invasion and metastasis [7]. The CD44+ CSCs are at least 10–200 fold enriched for tumorigenic properties in immunocompromised mice and were able to generate the heterogeneity of the original patient tumor [8]. PD-1/PD-L1 checkpoint blockade immunotherapy restores the immune system and is revolutionizing the therapeutic strategy of malignancies [9]. Recently, five immune checkpoint inhibitors blocking PD-1 or PD-L1 were approved by the US Food and Drug Administration (FDA) in the management of advanced BC, which represents new therapeutic opportunities [10].

PD-L1 is a co-inhibitory molecule expressed mainly on dendritic cells, macrophages, activated T and B cells, and tumor cells as well [11]. The PD-1 pathway is activated to attenuate tumor immunity and facilitate tumor progression [12]. High expression of PD-L1 has been regarded as a poor prognostic biomarker in several tumor tissues, and quite a few evidence shows a positive association between the metastatic ability of cancer cells and PD-L1 expression [13]. In some cancers, the improper regulation of signaling pathways or chromosomal alterations causes PD-L1 overexpression [14]. PD-L1 stabilization induced by TNF-α through p65/CSN5 activation on cancer cells led to immune system evasion [15]. However, besides of the dysfunctional tumor immunity, we know nothing about whether overexpressed PD-L1 has its direct effect on BC invasion/progression. Herein, we demonstrated that PD-L1 protein abundance is regulated by ATG7-autophagy with an ATG7-initiated positive feedback loop, and is a key ATG7 downstream mediator for the stem-like property, invasion, and tumorigenesis of human BCs.

Autophagy, as a double-edged sword, is responsible for degrading unnecessary or damaged proteins and cellular organelles in human cancers [16]. Several autophagy-related genes (ATG) are critical for autophagic flux. Autophagy-related gene 7 (ATG7), an E1-like activating enzyme, participates in the initiation of autophagy [17,18]. ATG7 overexpression has been found in N-butyl-N-(4-hydroxybutyl) nitrosamine (BBN)-induced mouse MIBCs and human BC tissues in our previous studies [19]. The overexpression of ATG7 promotes human BC cells abnormal growth behavior both in vitro and in vivo [19]. Moreover, the TET1/USP28/CD44/RhoGDIβ pathway has been demonstrated to be critical for the oncogenic role of ATG7 [20]. Here, we further discovered another important ATG7 downstream mediator PD-L1, which exerted its oncogenic role in stem-like property, tumorigenesis, and invasion of human BC cells.

Forkhead box (FOX) proteins characterized by the forkhead winged helix-turn-helix DNA binding domain, are conserved transcription factor family of proteins [21,22]. FOXO3a, a member of the FOXO subfamily, mediates a range of cellular processes including proliferation, apoptosis, DNA damage, tumorigenesis and cell cycle progression [23]. FOXO3a has been reported to repress MYC expression by directly targeting the transcriptional repressor MXI1-SRα [24]. FOXO3a upregulates Fas ligand by inhibiting miR-21 transcription, and finally initiates the apoptosis of human neuroblastoma cells [25]. Thus, FOXO3a dysregulation could contribute to cancer development by directly regulating its target genes expression. Herein, we identified that FOXO3a was the transcription factor mediating the transcription of miR-145, which in turn promoting *pd-l1* mRNA degradation, and finally decreased human BC cell stem-like properties. The function of FOXO3 has been reported to be regulated by post-transcriptional suppression, such as microRNAs (miRNAs), protein–protein interactions and post-translational modifications (PTMs) [26,27,28]. In the current study, we uncovered that ATG7 overexpression promoted autophagic removal of FOXO3a, in turn inhibiting miR-145 transcription, and further resulting in *pd-l1* mRNA stabilization and protein induction, finally promoted stem-like property, tumorigenesis, and invasion of human BCs.

## 2. Results

### 2.1. PD-L1 Was an ATG7 Downstream Mediator for Promoting Human High Invasive BC Cell Stem-Like Property, Invasion, and Anchorage-Independent Growth

It has been reported that PD-L1 expression levels are correlated with the response to PD-1-PD-L1 blockade in tumor cells [29,30]. Our previous studies have discovered that ATG7 overexpressed plays a critical role in cell invasion, growth and sphere formation of human BC cells [19,20].

**Figure 1:**
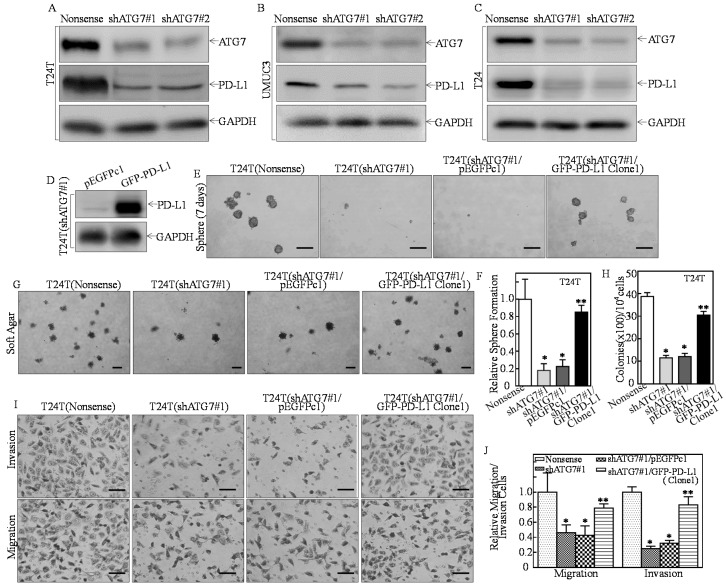
PD-L1 acted as an ATG7 downstream mediator being responsible for ATG7-promoted stem-like property, invasion, and anchorage-independent growth in human high invasive BC cells. (**A**–**C**) T24T, UMUC3, T24 cells were stably transfected with ATG7 knockdown constructs (#1 & #2), respectively. Western Blot was used to assess the ATG7 protein knockdown efficiency and its effects on other protein expression. (**D**) The GFP-tagged PD-L1 overexpression plasmid was stably transfected into T24T(shATG7#1) cells. (**E**,**F**) The indicated cells were subjected to determination of sphere formation abilities according to the manufacturer’s instruction, the number of spheroid formed cells were counted as described in the section of “Materials and Methods”. The asterisk (*) indicates a significant decrease in comparison to scramble nonsense transfectant (* *p* < 0.05), while the symbol (**) indicates a significant increase in comparison to T24T(shATG7#1/pEGFPc1) cells (** *p* < 0.05). (**G**,**H**) The indicated cells were subjected to anchorage-independent soft agar assay using the protocol described in the section of “Materials and Methods”. Representative images of colonies of indicated cells were photographed under an Olympus DP71 (**G**). The number of colonies was counted with more than 32 cells of each colony and the results were presented as colonies per 10^4^ cells, and the bars show mean ± SD from three independent experiments (**H**). The asterisk (*) indicates a significant decrease in comparison to scramble nonsense transfectant (* *p* < 0.05), while the symbol (**) indicates a significant increase in comparison to T24T(shATG7#1/pEGFPc1) cells (** *p* < 0.05). (**I**) Invasion abilities of the indicated cells were determined using BD BiocoatTM matrigelTM invasion chamber. The migration ability was determined using the empty insert membrane without the matrigel, while the invasion ability was evaluated using the same system except that the matrigel was applied. (**J**) The invasion ability was normalized to the insert control according to the manufacturer’s instruction. The asterisk (*) indicates a significant inhibition in comparison to T24T(Nonsense) cells (* *p* < 0.05), while the symbol (**) indicates a significant increase in comparison to T24T(shATG7#1/pEGFPc1) (** *p* < 0.05). Scale bars in (**E**,**I**) = 200 μm, Scale bars in (**G**) = 500 μm.

To test whether PD-L1 expression is responsible for ATG7′s promotion of tumorigenesis and stem cell-like properties of human BCs, we firstly transfected into highly invasive human BC cell lines with shATG7#1 and shATG7#2 (Figure 1A–C). Knockdown of ATG7 dramatically decreased PD-L1 protein expression (Figure 1A–C). To determine PD-L1′s biological contribution in ATG7 regulating stem-like property, invasion, and tumorigenesis, the GFP-PD-L1 constructs were stably transfected into T24T(shATG7#1) cells (Figure 1D). ATG7 knockdown almost completely abolished sphere formation in T24T cells (Figure 1E). In comparison to T24T(shATG7#1) cells, GFP-PD-L1 ectopic expression restored the sphere formation (Figure 1E,F), anchorage-independent growth (Figure 1G,H), migration and invasion of human BC cells (Figure 1I,J). These results demonstrated that PD-L1 is a novel ATG7 downstream regulated gene and plays a critical role in ATG7-mediated positive regulation of human high invasive BC cell stem-like property, invasion, and anchorage-independent growth.

### 2.2. ATG7 Promoted pd-l1 mRNA Stability by Regulating Its 3′-UTR Activity

**Figure 2:**
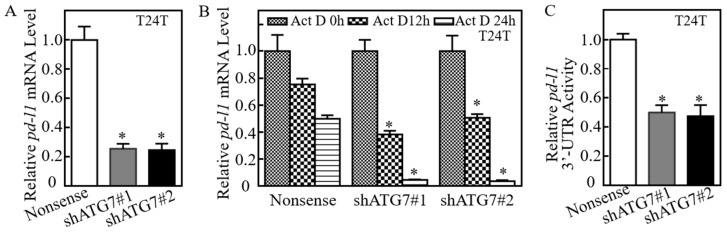

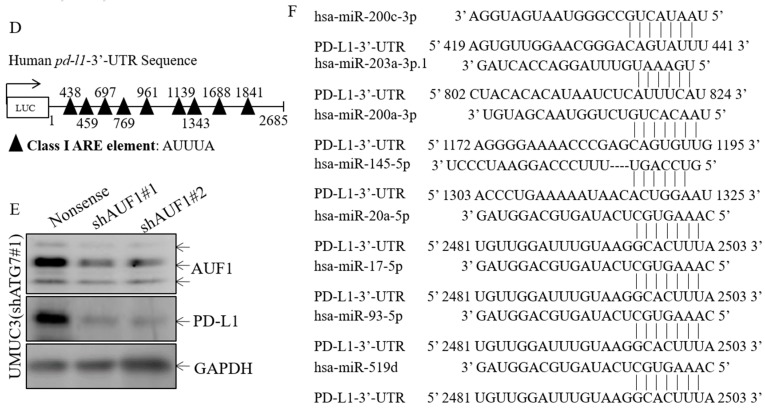
ATG7 inhibition destabilized *pd-l1* mRNA by attenuating its 3′UTR activity in human BC cells. (**A**) Real-time PCR was applied to compare the mRNA levels of *pd-l1* among T24T(shATG7#1), T24T(shATG7#2) and T24T(Nonsense) cells, and gapdh was used as internal control. (**B**) T24T(shATG7#1), T24T(shATG7#2), and T24T(Nonsense) cells were seeded into 6-well plates. After synchronization, the cells were used to determine *pd-l1* mRNA stability in the presence of Actinomycin D (Act D) by using Real-time PCR. *gapdh* was used as internal control. (**C**) The pMIR-*pd-l1* 3′-UTR reporter was transiently transfected into the indicated cells and luciferase activity of each transfectant was evaluated. The luciferase activity was presented as a relative to nonsense transfectant with normalized by using pRL-TK as an internal control. The bars show mean ± SD from three independent experiments. The symbol (*) indicates a significant decrease in T24T(shATG7) cells as compared to nonsense transfectant (* *p* < 0.05). (**D**) The predicted Class I ARE element on the 3′-UTR of *pd-l1* mRNA. (**E**) Cell lysates extracted from the indicated cells were subjected to Western Blot to determine AUF1, and PD-L1 protein expression, GAPDH was used as a protein loading control. (**F**) The potential microRNAs binding sites in 3′-UTR of *pd-l1* mRNA were analyzed by the TargetScan, Pictar and miRANDA database.

To elucidate the mechanisms of ATG7 promotion of PD-L1 protein expression, we first detected the effect of ATG7′s regulatory on *pd-l1* mRNA level. ATG7 knockdown remarkably inhibited *pd-l1* mRNA in T24T(shATG7) cells (Figure 2A). Therefore, we exploited the possibility of ATG7 stabilization of *pd-l1* mRNA. Upon treated with actinomycin D (Act D), *pd-l1* mRNA degradation rates in T24T(shATG7) cells was much faster than in T24T(Nonsense) cells (Figure 2B), revealing that ATG7 overexpression results in the stabilization of *pd-l1* mRNA in human BC cells. The 3-terminal untranslated region (3′-UTR) of mRNA has been reported to participate in its mRNA stability regulation [31]. Further results showed that knockdown of ATG7 significantly inhibited *pd-l1* mRNA 3′-UTR activity (Figure 2C), suggesting that ATG7 positively modulate 3′-UTR activity of *pd-l1* mRNA, which might be associated with ATG7 promotion of *pd-l1* mRNA stability in human BC cells. Moreover, the results from analyses of *pd-l1* mRNA 3′-UTR region showed that there were multiple ARE-elements in the *pd-l1* 3′-UTR sequence. Since our previous studies have reported that AUF1 protein is critical for ATG7 overexpression stabilizing *rhogdiβ* mRNA in human BC cells [32]. Thus, we knocked down AUF1 in UMUC3(shATG7#1) cells. The results indicated that AUF1 knockdown did not show a restoration of PD-L1 protein expression (Figure 2E), excluding the participation of AUF1 in ATG7 stabilization of *pd-l1* mRNA. MicroRNAs (miRNAs) are reported to regulate targeted gene expression by binding to mRNA 3′-UTR [33]. Putative miRNAs that could potentially target 3′-UTR of *pd-l1* mRNA were bioinformatics searched by using TargetScan [34], Pictar [35] and miRANDA [36] and the results were listed in Figure 2F. We anticipated that some of these miRNAs might be involved in the ATG7 regulation of *pd-l1* mRNA 3′-UTR activity.

### 2.3. ATG7 Overexpression Downregulated miR-145 and Subsequently Stabilized pd-l1 mRNA Through Directly Binding to Its 3′-UTR

**Figure 3:**
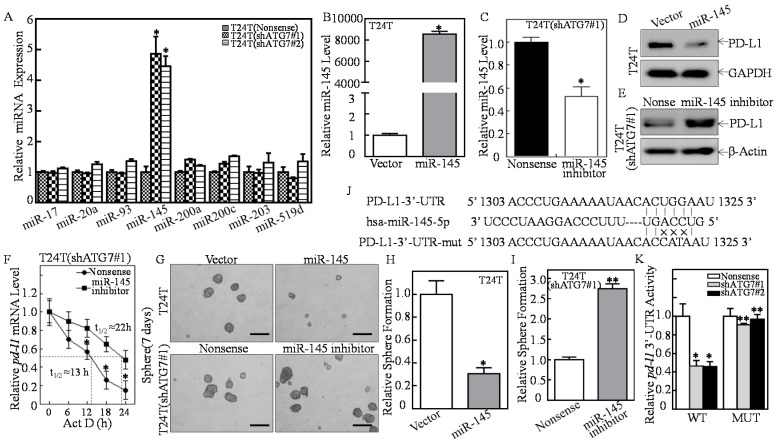
miR-145 was an ATG7 downstream regulator being responsible for stabilization of *pd-l1* mRNA via direct binding to its 3′-UTR. (**A**) Quantitative Real-time PCR was carried out to determine the expression of miRNAs in the indicated cells. The bars show mean ± SD from three independent experiments. The double asterisk (*) indicates a significant increase in comparison to T24T(Nonsense) cells (* *p* < 0.05). (**B**) miR-145 overexpression plasmids were stably transfected into T24T cells. The stable transfectants were identified by Real-time PCR. Bars represent mean ± SD from three independent experiments. Student’s *t*-test was utilized to determine the *p*-value and the asterisk (*) indicates a significant increase in comparison to scramble vector transfectant (* *p* < 0.05). (**C**) miR-145 inhibitor plasmids were stably transfected into T24T(shATG7#1) cells. The stable transfectants were identified by Real-time PCR. Bars represent mean ± SD from three independent experiments. Student’s *t*-test was utilized to determine the *p*-value and the asterisk (*) indicates a significant decrease in comparison to scramble vector transfectant (* *p* < 0.05). (**D**,**E**) Cell lysates extracted from the indicated cells were subjected to Western Blot for determining the protein expression of PD-L1. GAPDH was used as a loading control. (**F**) T24T(shATG7#1/miR-145) cells and T24T(shATG7#1/Nonsense) cells were seeded into 6-well plates. After synchronization, the cells were treated with Actinomycin D (Act D) for the indicated time points, then total RNA was isolated and subjected to Real-time PCR analysis for mRNA levels of *pd-l1*, and *gapdh* was used as an internal control. (**G**–**I**) The indicated cells were subjected to determination of sphere formation abilities according to the manufacturer’s instruction, the number of spheroid formed cells were counted as described in the section of “Materials and Methods”. The asterisk (*) indicates a significant decrease in comparison to Vector control transfectant (* *p* < 0.05), while the symbol (**) indicates a significant increase in comparison to T24T(shATG7#1/Nonsense) cells (** *p* < 0.05). (**J**) Schematic of the construction of the *pd-l1* mRNA 3′-UTR luciferase reporter and its mutants were aligned with miR-145. (**K**) Wild-type and mutant of *pd-l1* 3′-UTR luciferase reporters were transiently co-transfected with pRL-TK into the indicated cells, respectively. Luciferase activity of each transfectant was evaluated and the results were presented as relative *pd-l1* 3′-UTR activity. The bars show mean ± SD from three independent experiments. The double asterisk (**) indicates a significant restoration of 3′-UTR activity in mutant transfectant in comparison to mutant of WT *pd-l1* 3′-UTR luciferase reporter transfectant (**p < 0.05) of T24T(shATG7) cells in comparison to that of T24T(Nonsense) cells (* *p* < 0.05). Scale bars in (**G**) = 200 μm.

Based on the results shown in the above studies, Real-time PCR was performed to evaluate the miRNAs expressions as indicated in Figure 2F. As shown in Figure 3A, miR-145 was found to be the only one that was upregulated in T24T(shATG7) cells. To determine the potential effect of miR-145 on ATG7 regulation of PD-L1 expression, a construct expressing miR-145 or miR-145 inhibitor was transfected into T24T or T24T(shATG7#1) cells as shown in Figure 3B,C. MiR-145 ectopic expression resulted in a dramatical decrease of PD-L1 protein expression (Figure 3D), while miR-145 inhibition remarkably promoted PD-L1 protein expression (Figure 3E). These results revealed that miR-145 played an essential role in ATG7 promotion of PD-L1 expression. Further, miR-145 ectopic expression inhibited the sphere formation of T24T cells, while the inhibition of miR-145 in T24T(shATG7#1) cells markedly promoted the sphere formation and increased *pd-l1* mRNA stability (Figure 3F) in T24T(shATG7#1/miR-145 inhibitor) cells as compared with T24T(shATG7#1/Nonsense) (Figure 3G–I). To detect whether miR-145 could directly bind to *pd-l1* mRNA 3′-UTR, as indicated in Figure 3J, we constructed the miR-145 binding site mutation in *pd-l1* 3′-UTR-luciferase reporter. The results exhibited that *pd-l1* 3′-UTR luciferase activity was decreased in T24T(shATG7) cells, whereas the mutation luciferase reporter completely reversed this inhibition in ATG7 knockdown transfectant T24T(shATG7) (Figure 3K). Collectively, our results reveal that the direct binding of miR-145 to *pd-l1* 3′-UTR is important for miR-145 destabilization of *pd-l1* mRNA. Thus, ATG7 overexpression inhibits miR-145 expression, which subsequently binds to *pd-l1* 3′-UTR and results in *pd-l1* mRNA stabilization, and in turn enhancing human BC sphere formation.

### 2.4. ATG7 Inhibited miR-145 Transcription through Attenuating FOXO3a Protein Expression

**Figure 4:**
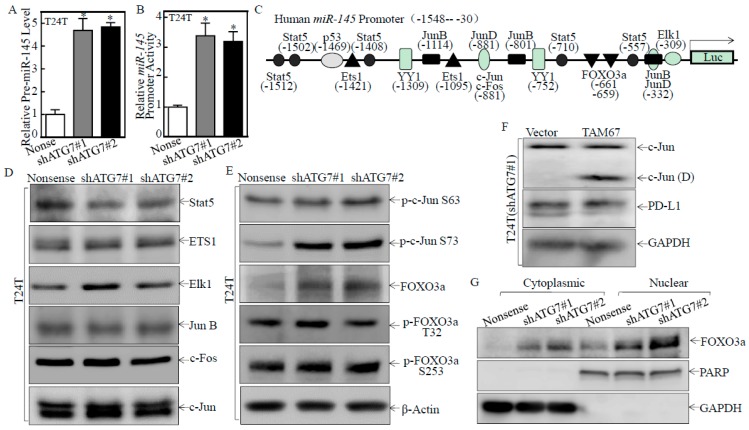
ATG7 overexpression inhibited *miR-145* mRNA transcription in human BC cells. (**A**,**B**) Pre-miR-145 expression and *miR-145* promoter transcription activity were evaluated in the indicated cells, the symbol (*) indicates a significant increase in comparison to scramble Nonsense transfectant (* *p* < 0.05). (**C**) Potential transcriptional factor binding sites in the *miR-145* promoter region (–1548 to –30) were analyzed by using the TRANSFAC 8.3 engine online. (**D**,**E**) The indicated cell extracts were subjected to Western Blot for determination of the expression of Stat5, ETS1, Elk1, JunB, c-Jun, p-c-Jun S63, p-c-Jun S73, FOXO3a, p-FOXO3a T32, and p-FOXO3a S253. β-Actin was used as a protein loading control. (**F**) Cell lysates extracted from T24T(shATG7#1/Vector) and T24T(shATG7#1/TAM67) cells were analyzed by Western Blot with a specific anti-c-Jun(D) antibody. (**G**) The indicated cell extracts were used to isolate cytoplasmic and nuclear fractions according to the protocol of the nuclear/cytosol fractionation kit. The isolated protein fractions were subjected to Western Blot. GAPDH and PARP were used as a control for cytosol or nuclear protein loading, respectively.

To explore the mechanisms of the ATG7 impairing miR-145, we tested the pre-miR-145 expression level and *miR-145* promoter activity. The results showed that both pre-miR-145 expression and *miR-145* promoter activity were markedly elevated in ATG7 knockdown transfectants (Figure 4A,B), indicating ATG7 regulation of miR-145 expression at the transcription level. Therefore, we bioinformatically analyzed and evaluated the expression of the potential transcriptional factors, which could bind to the -1548 to -30 region of the *miR-145* promoter (Figure 4C). Results indicated that knockdown of ATG7 consistently promoted FOXO3a protein expression and c-Jun phosphorylation at Ser63/73, while it did not show consistent effect on other transcription factors, such as Stat5, ETS1, ELK1, JunB and C-Fos (Figure 4D,E), suggesting that FOXO3a or c-Jun might be involved in ATG7 inhibition of miR-145 expression. To explore the potential role of c-Jun, dominant negative mutant Jun (TAM67) was stably transfected into T24T(shATG7#1) cells. As shown in Figure 4F, the introduction of TAM67 had no observable effect on PD-L1 protein expression, excluding the possible involvement of c-Jun in ATG7 promotion of PD-L1 expression. We further detected the effects of ATG7 knockdown on FOXO3a nuclear translocation by using a nuclear/cytosol fractionation assay. The results showed that knockdown of ATG7 resulted in the marked translocation of FOXO3a from cytoplasmic to nuclear in comparison to T24T(Nonsense) cells (Figure 4G), suggesting FOXO3a activation in ATG7 knockdown cells.

**Figure 5:**
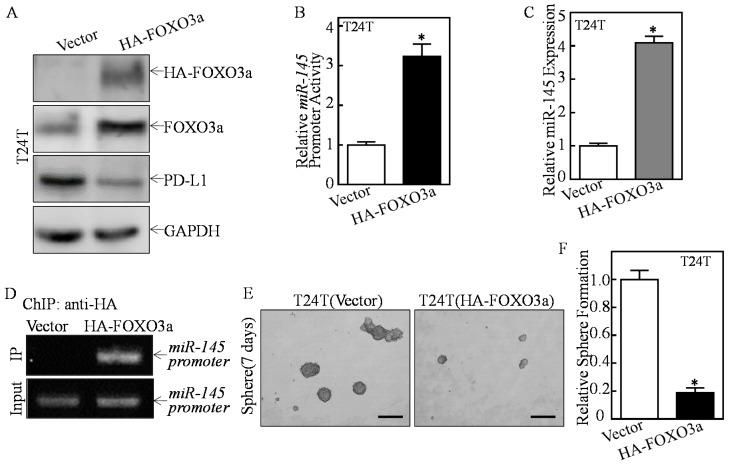
FOXO3a was an ATG7-regulated transcription factor mediating miR-145 downregulation in human BCs. (**A**) HA-tagged FOXO3a construct was stably transfected into T24T cells. The FOXO3a overexpression efficiency and its downstream PD-L1 expression were assessed by Western Blot. GAPDH was used as a protein loading control. (**B**,**C**) The *miR-145* promoter-driven luciferase reporter together with TK reporter was transfected into the indicated cells. Luciferase activity of each transfectant and miR-145 expression were evaluated and the bars show mean ± SD from three independent experiments. The asterisk (*) indicates a significant increase as compared with Vector transfectant (* *p* < 0.05). (**D**) 1 × 10^6^ T24T cells were seeded into 10-cm dish. After the cell density reached 80–90%, ChIP assay was performed with anti-FOXO3a antibody to determine FOXO3a binding to the motifs between −779 and −600 bp of the *miR-145* promoter. (**E**,**F**) The indicated cells were subjected to determination of sphere formation abilities according to the manufacturer’s instruction, the number of spheroid formed cells were counted as described in the Materials and Methods section. The asterisk (*) indicates a significant decrease in comparison to scramble Vector transfectant (* *p* < 0.05). Scale bars in (**E**) = 200 μm.

To detect whether FOXO3a was the transcription factor responsible for ATG7 inhibition of miR-145 transcription, we stably transfected HA-FOXO3a into T24T cells (Figure 5A). FOXO3a overexpression markedly promoted miR-145 promoter-driven transcription activity, and miR-145 expression, and inhibited PD-L1 protein expression (Figure 5A–C). Moreover, the chromatin immunoprecipitation (ChIP) assay was performed, and found that FOXO3a could directly bind to the *miR-145* promoter (Figure 5D). Consistently, the sphere formation ability was dramatically decreased in FOXO3a overexpressed T24T cells (Figure 5E,F). Collectively, these results strongly demonstrate that FOXO3a is the important transcription factor responsible for ATG7 inhibition of *miR-145* transcription and further promoted PD-L1 expression, as well as human BC stem-like property.

### 2.5. ATG7 Overexpression Promoted Autophagic Removal of FOXO3a in Human BC Cells

**Figure 6:**
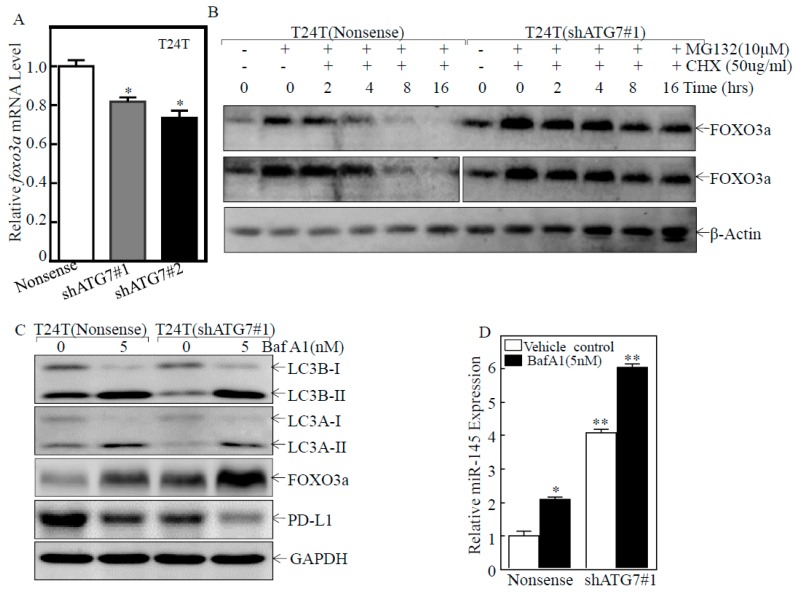
ATG7 promoted autophagic removal of FOXO3a protein. (**A**) Real-time PCR was used to detect *foxo3a* mRNA expression in the indicated cells. The asterisk (*) shows a significant inhibition in comparison to scramble nonsense transfectant (* *p* < 0.05). (**B**) FOXO3a protein stability was evaluated in the presence of cycloheximide (CHX) for the indicated times in T24T(shATG7#1) andT24T(shATG7#2) cells in comparison to that in T24T(nonsense) cells. (**C**,**D**) T24T(shATG7#1), T24T(shATG7#2), and T24T(nonsense) cells were treated with or without Baf A1 for 24 h, and the cell extracts were subjected to Western Blot to detect protein expression or Real-time PCR to determine miR-145 expression, as indicated. * *p* < 0.05; ** *p* < 0.05.

To elucidate how ATG7 inhibited FOXO3a protein expression, we assessed the effect of ATG7 on *foxo3a* mRNA abundance in T24T cells. ATG7 knockdown only showed a slight reduction of *foxo3a* mRNA as compared to its scramble nonsense transfectants (Figure 6A), suggesting that inhibition of FOXO3a protein by ATG7 might not occur at mRNA level. We further detected whether ATG7 regulated FOXO3a protein stability. The result showed that ATG7 knockdown remarkably increased FOXO3a protein stability (Figure 6B). Intriguingly, ATG7 plays an essential role in the process of autophagy, which delivers some proteins to lysosomes for degradation [37]. Thus, we next determined whether ATG7 overexpression mediated autophagy, and in turn promoting FOXO3a protein degradation. The results showed that knockdown of ATG7 did attenuate autophagy in T24T cells, while FOXO3a was markedly upregulated after inhibition of autophagy by Baf A1 treatment in comparison to the vehicle control (Figure 6C). Consistent with the alteration of FOXO3a protein, miR-145 expression was elevated in either ATG7 knockdown cells or autophagy inhibitor Baf A1-treated BC cells (Figure 6C,D). Consistently, PD-L1 protein expression was dramatically decreased in both ATG7 knockdown cells or autophagy inhibitor Baf A1-treated BC cells (Figure 6D). These results consistently reveal that ATG7 overexpression promotes autophagy, which further promotes FOXO3a protein degradation in human BC cells.

### 2.6. A Positive Feedback Loop was Formed by ATG7/autophagy, FOXO3a/miR-145 and PD-L1

**Figure 7:**
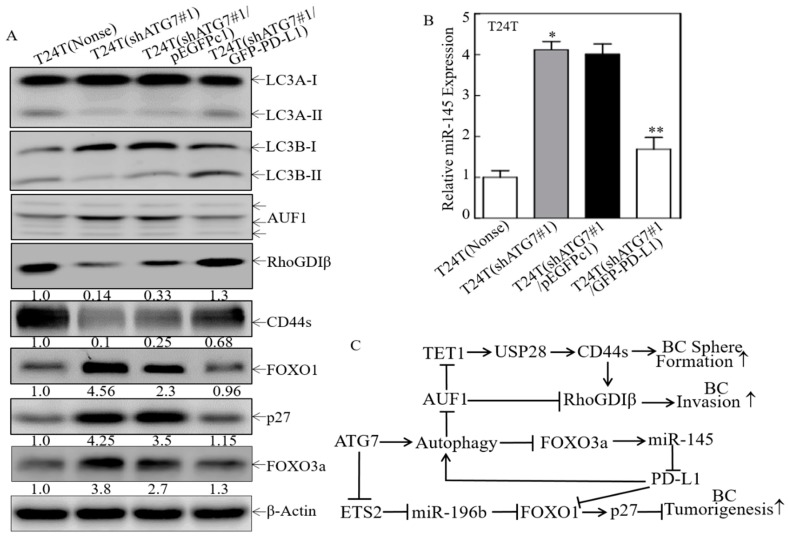
A positive feedback loop was formed to promote PD-L1 expression and ATG7/autophagy downstream effects in human BC cells. (**A**) T24T(shATG7#1) cells were stably transfected with the GFP-tagged PD-L1 overexpression plasmid, Western Blot was performed to determine the conversion of LC3 from LC3-I to LC3-II and the expression of AUF1, RhoGDIβ, CD44s, FOXO1, p27, and FOXO3a. β-Actin was used as a protein loading control. (**B**) Real-time PCR was performed to evaluate miR-145 expression in the indicated cells. * *p* < 0.05; ** *p* < 0.05. (**C**) The proposed mechanisms underlying ATG7 overexpression in the promotion of PD-L1 expression and human BCs stem-like property, invasion, and tumorigenesis.

As we have discovered that PD-L1 was an ATG7 downstream mediator for promotion of human high invasive BC cell invasion, anchorage-independent growth and sphere formation. We next tried to ask whether PD-L1 participated in the regulation of other ATG7 downstream effectors for human BC invasion/progression. The constructs that express GFP-PD-L1 were stably transfected into T24T(shATG7#1) cells. Ectopic expression GFP-PD-L1 in T24T(shATG7#1) cells promoted the conversion of LC3 from LC3-I to LC3-II, inhibited AUF1 and increased RhoGDIβ protein expression (Figure 7A), revealing PD-L1 was the critical upstream factor for ATG7-induced human BC invasion. Moreover, ectopic expression GFP-PD-L1 promoted CD44s (Figure 7A). Given our most recently have identified that the TET1/USP28/CD44/RhoGDIβ pathway that is crucial for ATG7′s oncogenic role in human BC cell invasion, metastasis and stem-like properties [20], our findings reveal that PD-L1 was the important upstream regulator for ATG7-induced human BC sphere formation and tumorigenesis. In addition, ectopic expression GFP-PD-L1 inhibited FOXO1 and p27 protein expression (Figure 7A), while our recent studies have indicated that ATG7 is able to promote BC tumorigenesis via ETS2/miR-196b/FOXO1/p27 axis [19]. Most importantly, we found that overexpression of PD-L1 in T24T(shATG7#1) cells attenuated FOXO3a protein expression and miR-145 expression as compared to T24T(shATG7#1/pEGFPc1) cells (Figure 7A,B). Collectively, our current studies together with our previous findings strongly reveal that a positive feedback loop was formed by ATG7/Autophagy and FOXO3a/miR-145 to regulate PD-L1 protein expression, and all of which together to form a network for the promotion of BC stem-like properties, tumorigenesis and invasion as illustrated in Figure 7C. Collectively, this study demonstrates that ATG7 autophagic removal of FOXO3a leads to attenuation of *miR-145* promoter transcription, and in turn resulting in the stabilization of *pd-l1* mRNA, further promoting stem-like property, invasion, and tumorigenesis of human high invasive BC cells. Furthermore, overexpression of PD-L1 in ATG7 knockdown cells reverses the defect of autophagy activation, FOXO3A degradation, and miR-145 transcription attenuation, suggesting a positive feedback loop to promote autophagy and consequently biological outcome due to ATG7/autophagy activation in human BC cells.

## 3. Discussion

In order to maintain cellular homeostasis, autophagy facilitates the turnover or clearance of misfolded, protein aggregates or long-lived proteins, and damaged organelles [38]. ATG7, a crucial protein for autophagic responses, has been found to be upregulated in BBN-induced basal MIBCs and human BC tissues, which plays an oncogenic role in BC stem-like property, invasion, lung metastasis and tumorigenic growth [19,20]. ATG7 has also been reported to facilitate the transcription of OCT4, promoting prostate tumor initiation, self-renewal, and drug resistance [39]. It has been reported that ATG5 or ATG7 knockdown reduces human hematopoietic stem/progenitor cells frequencies both in vitro and in vivo [40]. The current study identified that PD-L1 was a critical downstream mediator for ATG7 overexpression inducing stem-like property, invasion, and anchorage-independent growth of human BC. Our results discover a new linker among ATG7 upregulation, PD-L1 protein, human BC stem-like property and invasion, which offers novel insight into the therapeutic targeting of ATG7/PD-L1 for advanced human BCs.

Aberrant expression of PD-L1 suppresses cancer immunity and further promotes cancer progression in tumor cells [41]. Five immune checkpoint inhibitors blocking PD-1 or PD-L1 in the management of advanced bladder cancer have been approved by US FDA. PD-L1 overexpression has been shown to enhance cancer proliferation and invasion in vitro and promote cervical cancer growth in vivo [42]. However, the effect of high PD-L1 protein expression on tumor cell invasion has never been explored yet. Herein, we found that ectopic expression of GFP-PD-L1 promoted autophagy/AUF1/RhoGDIβ and autophagy/FOXO3a/miR-145 cascade signaling, elevated CD44a protein expression, impaired FOXO1 and p27 protein expression, all of which form a positive feedback loop to strengthen PD-L1 protein expression, further exhibiting the oncogenic role of ATG7 in human BCs stem-like property, invasion, and tumorigenesis. It has been reported that MAPK, PI3K/AKT, and Wnt/β-catenin pathways induce PD-L1 transcription by triggering the binding of the transcription factors to the *PD-L1* promoter [43]. EGFR activation induces EMT and myc-dependent PD-L1 expression in human salivary adenoid cystic carcinoma cells [44]. MiRNAs have been shown to target PD-L1 [45,46]. OCT4-activated miR-18a targets WNK2, PTEN, and SOX6 to repress p53 signaling or activate MEK/ERK, PI3K/AKT, and Wnt/β-catenin pathways to upregulate PD-L1 expression [42]. Our findings showed that ATG7 overexpression decreased miR-145 expression, in turn promoted *pd-l1 mRNA* stabilization by directly binding to its 3′-UTR. These results provide strong evidence demonstrating that miR-145 is a PD-L1 upstream regulator for mediating ATG7 elevation of BC stem-like property and invasion.

MiR-145 expression was decreased in multiple cancers including prostate, colon and lung, which is regarded as a tumor suppressor [47,48]. Multiple studies have focused on the role of miR-145 underlying carcinogenesis. MiR-145 reduces syndecan-1 and increases stem cell factors and induces cell differentiation and senescence on urothelial carcinoma cells [48]. MiR-145 could directly bind to TAGLN2 to suppress BC cell proliferation and migration [49] and silence c-Myc to inhibit the PTBP1/PKMs Axis, thereby reducing the cancer-specific energy metabolism [50]. Our previous study has discovered that isorhapontigenin (ISO) increases miR-145 expression, which inhibits SOX2 protein expression by directly binding to its 3′-UTR region. SOX2 inhibition results in the downregulation of cyclin D1 and suppression of anchorage-independent growth and sphere formation of patient-derived glioblastoma [51]. In this study, we discovered that ATG7 overexpression inhibits miR-145 expression, which further enhances the stability of *pd-l1* mRNA by directly binding to its 3′-UTR, and in turn promotes ATG7-induced stem-like property of human BCs. Mir-145 could be regulated at several levels to exert its tumor suppressor role. A sponge of miR-145, Circ_0058063 has been shown to regulate CDK6 expression and promote BC progression [52]. Reduced lncRNA-LET inhibited miR-145 biogenesis and subsequently led to the accumulation of CSCs [53]. Herein, FOXO3a was found to be responsible for the promotion of *miR-145* promoter transcription. Our finding further provides compelling evidence for miR-145′s tumor suppressor role in human BCs.

FOXO3a recruits transcriptional coactivators to trigger the transcription of downstream genes. FOXO3a plays crucial roles in apoptosis, cell cycle arrest, metabolism, and DNA repair, and participates in multiple pathological and physiological processes [54]. FOXO3 inhibits AGS cell growth by promoting Beclin1-medicated autophagy and inhibiting p62 protein expression in an acidic microenvironment [55]. XB130 promotes human colorectal cancer cell migration and invasion by enhancing phosphorylation of FOXO3a and epithelial-mesenchymal transition [56]. DFOG, a novel synthetic genistein analogue, decreases the level of phosphorylated FOXO3a to inhibit the ovarian cancer sphere-forming cell self-renewal activity [57]. In the current study, ATG7 overexpression inhibited FOXO3a protein expression and nuclear translocation, and further promoted stem-like properties of human high invasive BCs. A variety of cellular stimuli, including metabolic stress, growth factors, and oxidative stress tranduce signals to FOXO3a to regulate the downstream gene expression and protein-protein interactions [58,59]. Ubiquitination, phosphorylation, and acetylation have been reported to be involved in the regulation of these processes [59]. Crosstalk of several frequently deregulated signaling cascades: AKT, ERK/MAPK or IKK pathways, culminates in the inactivation of the tumor suppressive function of FOXO3a [60]. Here, we unexpectedly discovered that ATG7 exhibits autophagic removal role on FOXO3a protein, and promotes its protein degradation, and consequently inhibits FOXO3a-dependent transcriptional activity of its targeted gene miR-145.

In summary, this studies discovered a new feedback loop of ATG7/Autophagy/FOXO3a/ miR-145/PD-L1 pathway that acts as an ATG7 downstream factor being responsible for ATG7′s oncogenic role in the promotion of human BC cell stem-like property, invasion, and tumorigenesis. Specifically, ATG7 overexpression impairs FOXO3a protein expression, thereby decreases *miR-145* promoter transcription and its expression by directly binding to its promoter region. In turn, downregulated miR-145 reduces its direct binding to *pd-l1* mRNA3′-UTR and thereby stabilizes its mRNA and elevates PD-L1 protein expression, further resulting in promotion of human BC cell stem-like property and invasion. Besides, PD-L1 is defined to form a positive feedback loop to further enhance ATG7′s oncogenic role in human BC stem-like property, invasion, and tumorigenesis. Given the recent findings of the ATG7′s oncogenic effect on promoting BC progression, our new results provide the potential for developing a combination treatment with autophagy inhibitors and PD-1/PD-L1 immune checkpoint blockade therapeutic strategy for human BC patient treatment.

## 4. Materials and Methods

### 4.1. Plasmids, Reagents and Antibodies

Human *miR-145* promoter (from -1548 to -30) was cloned into the pGL3 Basic luciferase reporter to make a human *miR-145* promoter-driven luciferase reporter. The constructs of short hairpin RNA specific targeting ATG7 (shATG7), and AUF1(shAUF1) were purchased from Open Biosystems (Pittsburg, PA, USA). The GFP-tagged PD-L1 plasmid was constructed with Xho I and EcoR I using cDNA obtained from T24T cells. Human *pd-l1* 3′-UTR luciferase reporter was cloned into the pMIR luciferase assay vector. The HA-tagged FOXOA3a plasmid was obtained from Addgene (Cambridge, MA, USA). The miR-145 expression construct was kindly provided by Dr. Renato Baserga (Department of Cancer Biology, Thomas Jefferson University and Kimmel Cancer Center, Philadelphia, PA, USA) [61]. The miR-145 inhibitor was purchased from GeneCopoeia (San Diego, CA, USA). The dominant negative Jun plasmid (TAM67) was described and used in our previous studies [62]. Plasmids were prepared by the Plasmid Preparation/Extraction Maxi Kit from QIAGEN (Valencia, CA, USA). The chemical Actinomycin D was purchased from Calbiochem (Billerica, MA, USA). Proteasome inhibitor MG132 and protein synthesis inhibitor Cycloheximide (CHX) were purchased from Calbiochem. Bafilomycin A1 (Baf A1) was purchased from Santa Cruz (St. Louis, MO, USA). TRIzol reagent and the SuperScriptTM First-Strand Synthesis system were acquired from Invitrogen (Grand Island, NY, USA). PolyJetTM DNA in Vitro Transfection Reagent was purchased from SignaGen Laboratories (Rockville, MD, USA). The nuclear/cytosol fractionation kit was obtained from Biovision Incorporated (Milpitas, CA, USA).

The antibodies specific against ATG7, FOXO3a, p-FOXO3a T32, p-FOXO3a S253, FOXO1, p27, CD44, LC-3A, LC-3B, HA, PARP, c-Jun, p-c-Jun Ser63, p-c-Jun Ser73, c-Jun(D), JunB, Elk1, ETS1, Stat5, and GAPDH, were purchased from Cell Signaling Technology (Beverly, MA, USA). Antibodies against PD-L1, RhoGDIβ and β-Actin, were bought from Santa Cruz (Dallas, TX, USA). The antibody specific against AUF1 was purchased from Aviva Systems Biology (San Diego, CA, USA).

### 4.2. Cell Lines and Cell Culture

T24 and T24T human BC cell lines were described and used in our previous studies [19,63]. These cells were cultured in DMEM-F12 (1:1) (Invitrogen, Grand Island, NY, USA), supplemented with 2 μM L-glutamine, 5% heat-inactivated FBS, and 25 μg/mL gentamycin. Human BC cell lines, UMUC3, were maintained in DMEM (Invitrogen), supplemented with 2 μM L-glutamine, 10% heat-inactivated FBS, and 25 μg/mL gentamycin. All cell lines were subjected to DNA tests and authenticated before/after utilization for research by Genetica DNA Laboratories (Burlington, NC, USA) using a PowerPlex^®^ 16 HS System.

### 4.3. Western Blot Analysis

Western blot was performed in our previous publication [64]. T24T, T24, UMUC3 cells and their transfectants were seeded in 6-well plates and cultured in the normal medium until 70–80% confluence. Following culture cells in 0.1% FBS medium for 12 h, the medium were replaced with 5% FBS DMEM-F12 (1:1) or 10% FBS DMEM for another 12 h. Whole cell extracts were prepared with the cell lysis buffer (10 mM Tris-HCl, pH 7.4, 1% SDS, and 1 mM Na_3_VO_4_) and then were subjected to Western Blot analysis. The images were captured by scanning with the phosphorimager (Typhoon FLA 7000, GE, Pittsburgh, PA, USA).

### 4.4. Luciferase Report Assay

Luciferase reporter assays were carried out as described as previously [65].

### 4.5. Semiquantitative or Quantitative RT–PCR

Semiquantitative or quantitative RT–PCR was carried out to examine the mRNA or miRNA expression, as previously described [19,62], and the primers were described in Table S1.

### 4.6. The Construct of pd-l1 3′-UTR Mutant Luciferase Reporters

A three-point mutation was introduced into the seed region of *miR-145/pd-l1* putative interacting sequence (see Figure 3I) using primers MIRMUTFOR, 5′-*ACC CTG AAA AAT AAC ACC ATA ATT CCT TTT CTA GCA T*-3′ and MIRMUTREV, 5′-*ATG CTA GAA AGG AAT TAT GGT GTT ATT TTT CAG GGT*-3′, according to the site-directed mutagenesis protocol (Quick-Change Site-Directed Mutagenesis, Stratagene, Santa Clara, CA, USA), for producing the pMIR-*pd-l1* 3′-UTR mutant plasmid. Constructs were all sequence verified by GENEWIZ (South Plainfield, NJ, USA).

### 4.7. ChIP Assay

ChIP assay was carried out as described in our previous publication [65]. To specifically amplify the region containing the putative responsive elements on the human *miR-145* promoter, PCR was performed with the following pair of primers: 5′-*AAG CAC AAT GAG CAG AGG AGA G*-3′ and 5′-*TTA GGG TAC AAT CTC AGC TCA GCA C*-3′. The PCR products were separated on 2% agarose gels and stained with ethidium bromide; the images were then scanned with a UV light.

### 4.8. Sphere Formation Assay

The indicated cells were cultured in a 6-well ultra-low attachment culture plate for 5 or 7 days, and Olympus DP71 microscopy (Olympus America Inc., Center Valley, PA, USA) was used to capture the images, and the number of spheroids formed cells in each image was counted by the“Image J” software [66] for at least 6 random images. The results presented as the percentage of the spheroid colonies.

### 4.9. Cell Migration and Invasion

As described in our earlier publication, cell migration and invasion assay were performed [64]. Control inserts without matrigel and permeable support for 24 well plate with 8.0 μm transparent PET membrane were purchased from Corning Incorporated (Corning, NY, USA) (353097), the invasion kit (354480) was purchased from BD Biosciences (Bedford, MA, USA). The invasion assay was performed in normal cell culture serum according to the manufacturer’s instruction. The images were taken under microscopy, Olympus DP71 (Olympus America Inc. Center Valley, PA, USA), and the number of the cells in each image was counted by the Image J software. According to the manufacturer’s instruction, the invasion rate was normalized with the insert control.

### 4.10. Anchorage-Independent Growth Assay

As described in our previous studies, anchorage-independent growth (soft agar assay) was carried out [67]. Briefly, the 1 × 10^4^ cells in 10% FBS Basal Medium Eagle (BME) containing 0.33% soft agar and were seeded over the basal layer containing 0.5% agar containing 10% FBS BME in each well of 6-well plates. The plates were incubated in 5% CO_2_ incubator at 37 °C for 3 weeks. Colonies were captured under a microscope and only colonies with over 32 cells were counted. The results were presented as mean ± SD obtained from three independent experiments.

### 4.11. Statistical Analysis

Prism 5.0 Software (GraphPad Software, San Diego, CA, USA) was used to perform the statistical analysis. All data are shown as the means of triplicate assays ± SD. Student’s *t*-test was employed to detect the significance of differences between various groups. The differences were considered significant at *p* < 0.05.

## 5. Conclusions

The identification of the feedback loop of ATG7/Autophagy/FOXO3a/miR-145/PD-L1 pathway provides an important insight into understanding the oncogenic role of ATG7 in the promotion of human BC cell stem-like property, invasion, and tumorigenesis, suggesting that autophagy inhibitors and PD-1/PD-L1 immune checkpoint blockade maybe combined to be a potential therapeutic strategy for the treatment of human BC patients.

## Supplementary Materials

The following are available online at https://www.mdpi.com/2072-6694/11/3/349/s1, Table S1: The primers used in this project.
