# Correction: Zhu et al. A Feedback Loop Formed by ATG7/Autophagy, FOXO3a/miR-145 and PD-L1 Regulates Stem-like Properties and Invasion in Human Bladder Cancer. *Cancers* 2019, *11*, 349

**DOI:** 10.3390/cancers15051403

**Published:** 2023-02-23

**Authors:** Junlan Zhu, Yang Li, Yisi Luo, Jiheng Xu, Huating Liufu, Zhongxian Tian, Chao Huang, Jingxia Li, Chuanshu Huang

**Affiliations:** Nelson Institute of Environmental Medicine, New York University School of Medicine, New York, NY 10010, USA

## Error in Figure

In the original article [1], there was a mistake in Figure 1E (right panel) as published. A sphere image was incorrectly used in Figure 1E (right panel). The corrected Figure 1E is shown below.

The authors apologize for any inconvenience caused and state that the scientific conclusions are unaffected. The original article has been updated.

## Figures and Tables

**Figure 1:**
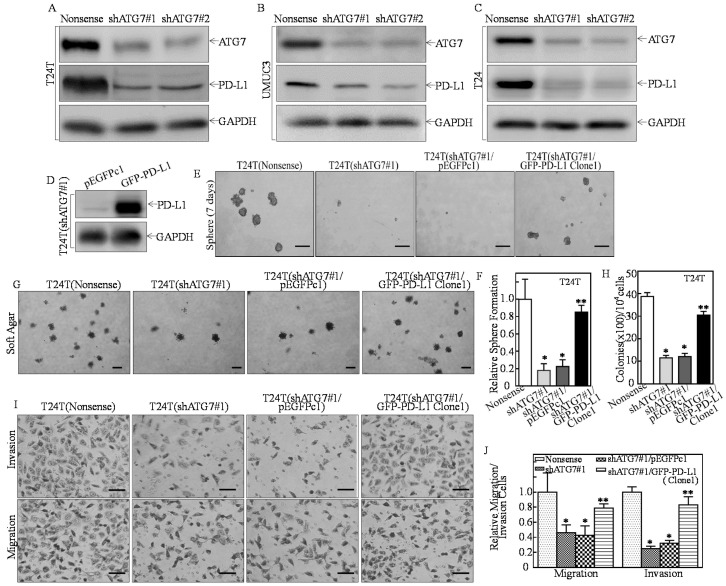
PD-L1 acted as an ATG7 downstream mediator being responsible for ATG7-promoted stem-like property, invasion, and anchorage-independent growth in human high invasive BC cells. (**A**–**C**) T24T, UMUC3, T24 cells were stably transfected with ATG7 knockdown constructs (#1 & #2), respectively. Western Blot was used to assess the ATG7 protein knockdown efficiency and its effects on other protein expression. (**D**) The GFP-tagged PD-L1 overexpression plasmid was stably transfected into T24T(shATG7#1) cells. (**E**,**F**) The indicated cells were subjected to determination of sphere formation abilities according to the manufacturer’s instruction, the number of spheroid formed cells were counted as described in the section of “Materials and Methods”. The asterisk (*) indicates a significant decrease in comparison to scramble nonsense transfectant (* *p* < 0.05), while the symbol (**) indicates a significant increase in comparison to T24T(shATG7#1/pEGFPc1) cells (** *p* < 0.05). (**G**,**H**) The indicated cells were subjected to anchorage-independent soft agar assay using the protocol described in the section of “Materials and Methods”. Representative images of colonies of indicated cells were photographed under an Olympus DP71 (**G**). The number of colonies was counted with more than 32 cells of each colony and the results were presented as colonies per 10^4^ cells, and the bars show mean ± SD from three independent experiments (**H**). The asterisk (*) indicates a significant decrease in comparison to scramble nonsense transfectant (* *p* < 0.05), while the symbol (**) indicates a significant increase in comparison to T24T(shATG7#1/pEGFPc1) cells (** *p* < 0.05). (**I**) Invasion abilities of the indicated cells were determined using BD BiocoatTM matrigelTM invasion chamber. The migration ability was determined using the empty insert membrane without the matrigel, while the invasion ability was evaluated using the same system except that the matrigel was applied. (**J**) The invasion ability was normalized to the insert control according to the manufacturer’s instruction. The asterisk (*) indicates a significant inhibition in comparison to T24T(Nonsense) cells (* *p* < 0.05), while the symbol (**) indicates a significant increase in comparison to T24T(shATG7#1/pEGFPc1) (** *p* < 0.05). Scale bars in (**E**,**I**) = 200 μm, Scale bars in (**G**) = 500 μm.
