# Supplementary figures and images for: A Feedback Loop Formed by ATG7/Autophagy, FOXO3a/miR-145 and PD-L1 Regulates Stem-Like Properties and Invasion in Human Bladder Cancer
Source: Cancers (Basel). 2019 Mar 12;11(3):349. doi: 10.3390/cancers11030349 (PMC6468999; doi:10.3390/cancers11030349)

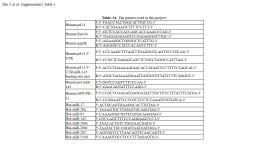

Supplement: Supplementary file 1 [file cancers-11-00349-s001.zip › cancers-436120-supplementary/docProps/thumbnail.jpeg]
